# Effect of chemical surface pretreatments on microtensile bond strength of resin composite to dentin when using universal adhesives: a systematic review of laboratory studies

**DOI:** 10.1038/s41405-026-00484-3

**Published:** 2026-10-20

**Authors:** Ereny Isaac, Safaa Helmy Abd-Elhakim, Hamdi H. Hamama, Mona Riad

**Affiliations:** 1 Conservative Dentistry Department, Faculty of Dentistry, Minia University, Minia, Egypt; 2 Conservative Dentistry Department, Faculty of Dentistry, Mansoura University, Mansoura, Egypt; 3 Conservative Dentistry Department, Faculty of Oral and Dental Medicine, Delta University for Science and Technology, Gamasa, Egypt; 4 Conservative Dentistry Department, Faculty of Dentistry, Cairo University, Cairo, Egypt

**Keywords:** Restorative dentistry, Dental materials, Dental treatments

## Abstract

**Objective:**

To evaluate the effect of different chemical pretreatments of sound dentin on the microtensile bond strength (µTBS) of resin composite to dentin.

**Methods:**

A comprehensive online search was performed across four electronic databases: PubMed (Medline), Web of Science, Scopus, and the Cochrane libraries to identify relevant studies, followed by screening based on established eligibility criteria. In vitro studies examining the effect of chemical pretreatment on the microtensile bond strength of sound dentin to resin composite were included. Only English studies published from 2013 until February 10, 2026 were considered. Studies that used animal teeth, focused on carious, sclerotic, or desensitized dentin, or assessed mechanical or laser pretreatments were excluded. The protocol followed PRISMA guidelines and was registered with PROSPERO (CRD420261299181). Selected studies underwent a risk-of-bias analysis.

**Results:**

A total of 17 studies met the inclusion criteria. Across the included studies, microtensile bond strength results revealed that certain chemical pretreatments can significantly enhance immediate bond strength as well as maintain long-term bond durability. Pretreatment with 50% Dimethyl Sulfoxide (DMSO), acetone wet-bonding, glutaraldehyde desensitizers, and copper-based (Cu-P) pretreatments significantly increased µTBS of the majority of dental adhesives and preserved their long-term stability. Selective dentin etching (SDE) for 3 seconds, along with glycolic acid and zirconium oxynitrate (ZON) etchants, yielded significantly higher immediate bond strengths than traditional 15-second phosphoric acid etching. In cases of blood contamination, grape seed extract and hesperidin collagen cross-linkers restored bond strength to nearly uncontaminated control levels. Conversely, silver-based solutions significantly reduced µTBS, and limonene had no significant effect on bond strength.

**Conclusion:**

Several chemical pretreatments, including selective dentin etching, solvent-based pretreatments, copper-based (Cu-P) pretreatments, glutaraldehyde desensitizers, and collagen cross-linkers, enhanced the bond strength of universal adhesives to sound dentin.

## Introduction

In the past few decades, adhesive dentistry has quickly grown; however, maintaining long-term durability of the resin-dentin interface has always been a major clinical challenge. The primary factor contributing to replacement of dental restorations is interfacial biodegradation. It has been reported that more than 50% of all dental work involves replacing old fillings [1–3]. This process mainly occurs when the depth of monomer infiltration does not comply with the depth of demineralization, leaving exposed collagen fibrils that are susceptible to enzymatic degradation over time. Such degradation allows bacteria and their toxins to penetrate, leading to secondary caries [3,4]. Dentin surface pretreatment before bonding is therefore essential to modify or remove the smear layer, optimize substrate wettability, and incorporate agents that improve collagen quality, thereby enhancing dentin bonding and preventing bond degradation [5,6].

Cysteine cathepsins and matrix metalloproteinases (MMPs) are key endogenous proteases that contribute to collagen degradation. MMPs 2, 3, 8, 9, and 11 are present in dentin [7]. These enzymes mainly rely on zinc and calcium [6]. Although they stay inactive in mineralized dentin, they can be triggered by low pH levels. Such acidity may result from acids used in bonding procedures, acids produced by cariogenic bacteria, hemostatic agents, or very acidic universal adhesives [8–10].

Moreover, water plays a central role in bond degradation. While moisture is necessary to keep the collagen matrix expanded for resin infiltration, excess water can cause phase separation of adhesive components and hinder the formation of cross-linked polymer chains in the hybrid layer, thereby compromising mechanical properties. Additionally, residual water in the collagen matrix allows endogenous enzymes to degrade demineralized collagen. Bond instability is often exacerbated during the bonding process when excessive air-drying of the etched dentin causes collagen shrinkage and collapse, making it difficult for adhesive monomers to properly wet the conditioned surface [11,12].

Given these challenges, a wide range of dental adhesives and bonding techniques have emerged. The introduction of “universal adhesives” was a significant development in this field. This new generation of adhesives is extremely adaptable, as they can be effectively applied using either an etch-and-rinse (ER) or self-etch (SE) strategy [3]. However, previous studies have reported conflicting findings when comparing the effectiveness of these two bonding methods [12]. While some meta-analyses and laboratory reports state that phosphoric acid pre-etching enhances dentin bond strength [12], other investigations show no significant differences in bonding outcomes between the ER and SE modes [3,5]. These discrepancies are primarily attributed to variations in the chemical composition of universal adhesives, such as pH, solvent types, and functional monomer formulations, which dictate their overall sensitivity to substrate moisture and long-term hydrolytic stability [3,12,13].

Several attempts, with varying degrees of success, have been made to address these limitations. This review focused on chemical pretreatments that shared the common goal of improving bond durability. These treatments include solvent-based strategies that employ agents such as Dimethyl Sulfoxide (DMSO), acetone, and ethanol to enhance surface wettability, re-expand collapsed collagen, and promote the deep infiltration of hydrophobic monomers into the demineralized matrix [2,3,12]. Collagen stabilization strategies act by biologically altering the dentin substrate via matrix metalloproteinase (MMP) inhibitors and collagen cross-linkers, which include natural agents such as resveratrol, epigallocatechin-3-gallate (EGCG), grape seed extract (GSE), hesperidin, and limonene [4,6,10,14] as well as synthetic agents like 2% chlorhexidine and glutaraldehyde [13,15]. These agents are applied to dentin as a pretreatment to reinforce and stabilize collagen against degradation.

A promising copper-based pretreatment markedly enhances bond strength by inducing the in situ formation of copper phosphate nanoparticles within the demineralized dentin matrix. These nanoparticles reinforce the demineralized collagen network, preventing its collapse and promoting deeper infiltration of adhesive monomers. Copper also acts as an indirect collagen cross-linking agent and a potent inhibitor of matrix metalloproteinases (MMPs), thereby reducing biological degradation of the hybrid layer [5,16].

Finally, alternative conditioners, such as glycolic acid [17] and metal salt-based conditioners, specifically zirconium oxynitrate (ZON) [18], have been proposed to provide a less aggressive etching pattern than traditional phosphoric acid. These strategies aim to provide a more stable interface than the classic etch-and-rinse mode. Unlike phosphoric acid, which deeply demineralizes dentin and leaves collagen vulnerable, these conditioners create micro-mechanical interlocking while maintaining more Hydroxyapatite (HAp) for additional chemical binding of the 10-MDP functional monomer present in some universal adhesives, which is totally absent when dentin is etched with phosphoric acid [18].

Despite the growing number of studies examining these pretreatments, published results remain inconsistent owing to discrepancies in experimental protocols, adhesive systems, and aging methods, hence creating a research gap. In addition, previous systematic reviews have assessed the bonding performance of universal adhesives, either by examining a broad range of pretreatment strategies or by targeting specific factors, such as the effect of etching [19–21]. In contrast, this review focuses specifically on chemical dentin pretreatments and their influence on microtensile bond strength to sound dentin. Accordingly, a thorough comparison of summarized data, derived from a systematic review of the available evidence, is essential. Therefore, this systematic review of in vitro studies aimed to evaluate and compare the effect of different chemical dentin pretreatments on the microtensile bond strength of resin composite to sound dentin. Specifically, it addresses the research question: “ Does chemical surface pretreatment of dentin prior to the application of universal adhesives affect bond strength compared with no dentin pretreatment?”

## Methods

The present systematic review was performed in compliance with the Preferred Reporting Items for Systematic Reviews and Meta-Analyses (PRISMA) Checklist [22]. In addition, the review protocol was recorded in the International Prospective Register of Systematic Reviews (PROSPERO) bearing the number CRD420261299181.

### Eligibility criteria

The studies were chosen based on the PICO methodology: Population (P): human molars with sound dentin. Intervention (I): chemical surface pretreatment before applying universal adhesives for resin composite. Comparison (C): Comparison with untreated dentin surfaces. Outcome (O): bond strength of resin composite to sound dentin, assessed by microtensile bond strength (μTBS) testing. Only English-language in vitro studies published from 2013, corresponding to the introduction of universal adhesives, until February 10, 2026, were included. Based on the specified inclusion criteria, the following were excluded: studies involving animal teeth; studies focused on carious, sclerotic or desensitized dentin only when no separate experimental group using sound dentin was available; review articles; clinical trials; case reports; studies on enamel; and studies unrelated to dentistry or the research question. Furthermore, studies assessing mechanical or laser pretreatments were also omitted.

In this review, chemical dentin pretreatments were defined as agents or solutions applied to the dentin surface before universal adhesive application to modify the dentin substrate or its surface properties. The Results section presents the pretreatment agents and categories identified during data extraction from the included studies.

### Data source and search strategy

A literature search was performed electronically using the following databases: PubMed (including Medline), Scopus, Web of Science, and Cochrane Library. To guarantee that no relevant research was overlooked throughout the search, a manual search of grey literature was also performed. Details of the search strategy, Boolean operators, and keywords utilized in each database are available in Supplementary File 1. The chosen articles were imported into the reference manager software “Zotero” (Version 7.0.30) after the search was finished for duplicate detection and removal.

### Study selection

All studies were evaluated against the eligibility criteria. Studies retrieved from several databases were exported to the Rayyan platform (www.rayyan.ai). The screening process was completed in two steps: (1) reviewing titles and abstracts to assess relevance, and (2) reviewing the full texts of studies that appeared eligible. These full-text evaluations determined the final selection of articles. A third reviewer settled disagreements between reviewers by providing the definitive decision.

### Data extraction

Specific data were extracted from each study and analyzed descriptively, and essential variables were tabulated, including study identification details (author(s), year of publication) and study characteristics (tooth type, sample size, type of chemical pretreatment, comparators, adhesive systems used, and testing time or aging periods). The extracted data from the studies were summarized and presented in Table 1.

**Table 1:** Characteristics of included studies.

Authors (Year)	Tooth type	Sample size Number of teeth (per group)	Chemical Intervention/ Pretreatment	Comparator	Testing time/ ageing	Adhesive systems
**Stape, T.H.S. et al. (2018)** [11]	Sound human molars	96 (8)	Dimethyl Sulfoxide pretreatments DMSO/ethanol or DMSO/H2O in wet or dry conditions	Untreated dentin surfaces	Immediate testing; storage in distilled water at 37 °C for 24 h	The study used 2 adhesive systems: **1-Scotchbond Universal Adhesive (3 M/ESPE)** **2-Adper Scotchbond Multi-Purpose (3 M/ESPE)** A three-step etch-and-rinse adhesive
**Cecchin, D. et al. (2018)** [17]	Human third molars (for dentin evaluation); human central incisors (for enamel evaluation)	96 (8) (for dentin samples)	35% α-hydroxy glycolic acid (GA) as an alternative surface etchant for dentin	Standard Etchant Control: 35% phosphoric acid (PA)	Immediate testing: storage in distilled water at 37 °C for 24 h	The study used 3 adhesive systems: **1-Scotchbond Universal Adhesive** 3 M ESPE (St Paul, MN, USA) **2- One Step Plus** Bisco (Schaumburg, IL, USA) **3- Adper Single Bond** 3 M ESPE (St Paul, MN, USA) An etch-and-rinse adhesive system
**Zhang, Z. et al. (2020)** [14]	Sound human molars	30 (6)	Pretreatment with epigallocatechin-3-gallate (EGCG) incorporated into a 50% (v/v) dimethyl sulfoxide (DMSO) aqueous solution	Two control groups: 1- Water Wet-Bonding (WWB): This served as the primary control, without any chemical additives 2- DMSO Wet-Bonding (DWB): DMSO aqueous solution alone (without EGCG) to isolate the effects of the solvent from the antioxidant/inhibitor	Immediate testing after 24 h storage in deionized water at 37 ◦C and After 1-month of collagenase aging and water aging	**Single bond Universal adhesive** (3 M ESPE, St. Paul, MN, USA)
**Peng, W. et al. (2020)** [4]	Sound human molars	36 (9)	Pretreatment with resveratrol/ethanol solutions as a multifunctional dentin primer	Ethanol primer only, without any resveratrol added	1. Immediate Group (water storage at 37 °C for 24 hours) 2. Thermocycling aging (10,000 thermal cycles) 3. Collagenase Aging (for one month)	**Scotchbond Universal Adhesive** (3 M ESPE, St. Paul, MN, USA)
**Fernandes, G.L. et al. (2020)** [15]	Sound human molars	20 (5)	Preapplication of 2% chlorhexidine gluconate	Untreated dentin surfaces	Immediate testing storage in distilled water at 37 °C for 24 h	**Single bond Universal adhesive** (3 M ESPE, St. Paul, MN, USA)
**Ahmed, M. et al. (2022)** [18]	Sound human molars	24 (8)	Dentin conditioned with an experimental metal salt-based conditioner (‘ZON’; Ivoclar Vivadent)	Two control groups: 1- Classic Etch-and-Rinse (E&R) Control 2- Self-Etch (SE) Control	1. Immediate Group (water storage at 37 °C for one week) 2. Thermocycling (50,000 thermocycles)	**Adhese Universal (AU)** (Ivoclar Vivadent)
**Mancuso, E. et al. (2023)** [13]	Sound human molars	48 (8)	Dentin pretreatment with glutaraldehyde-based desensitizers	Untreated dentin surfaces	aged in artificial saliva at 37 ◦C for 24 h (T0) or 12 months (T12)	The study used 2 adhesive systems: **1-Adhese Universal (AU)** (Ivoclar Vivadent) **2-iBond Universal (IBU)** (Kulzer GmbH)
**Pan, Y. et al. (2023)** [16]	Sound human molars	90 (10)	Copper-based (Cu-P) pretreatment	Acid-etched dentin surface, without receiving any chemical pretreatment	After storage in artificial saliva (pH 6.8) at 37 °C for 1 day	The study used 3 adhesive systems: **1- Single Bond Universal (SBU)** (3 M ESPE) **2- Prime Bond Universal (PBU)** (Dentsply Sirona, Konstanz, Germany) **3- Single Bond 2 (SB2)** (3 M ESPE, St. Paul, MN, USA) two-step etch-and-rinse adhesive
**Zhao, S. et al. (2023)** [2]	Sound human molars	60 (15)	The use of acetone as a dentin pretreatment solution following acid etching	Two control groups: 1- Water Wet-Bonding (WWB) 2- Ethanol Wet-Bonding (EWB)	1. Immediate Group (water storage at 37 °C for 24 hours) 2. Thermocycling aging (10,000 thermal cycles) 3. Collagenase Aging (for one month)	**Singlebond Universal adhesive** (3 M ESPE, St. Paul, MN, USA)
**Manihani, A.K. et al. (2023)** [6]	Sound human molars	36 (9)	The use of collagen cross-linkers (CCLs): 1- Grape Seed Extract (GSE): This extract contains proanthocyanidins (PAs) 2- Hesperidin Extract To the dentin, which was contaminated with blood and a hemostatic agent	Two control groups: 1- Uncontaminated clean dentin 2- positive control: Contaminated dentin with blood and a hemostatic agent	Immediate testing storage in distilled water at 37 °C for 24 h	**Singlebond Universal adhesive** (3 M ESPE, St. Paul, MN, USA)
**Karadas, M. et al. (2023)** [12]	Sound human molars	128 (8)	dentin pretreatment with a 50% dimethyl sulfoxide (DMSO) aqueous solution	Untreated dentin surfaces	1. Immediate Group (water storage at 37 °C for 24 hours) 2. Thermocycling aging (15,000 thermal cycles)	The study used 4 adhesive systems: **1- Scotchbond Universal (SBU)** (3 M ESPE, St Paul, MN, USA) **2- Clearfil Universal Bond (CUB)** (Kuraray Noritake, Okayama, Japan) **3- Ambar Universal Bond (AUB)**(FGM, Brazil) **4- G-Premio Bond (GPB)** (GC Corp., Tokyo, Japan)
**D’ Alessandro, C. et al. (2024)** [28]	Sound human molars	60 (10)	Dentin pretreatment with one of two silver-based solutions, followed by potassium iodide (KI), before applying the adhesive 1- Ammonia-Based Solution (Riva Star): This is a 38% silver diamine fluoride (SDF) 2- Water-Based Solution (Riva Star Aqua): A patented aqueous solution of silver fluoride (AgF)	Untreated dentin surfaces	(T0) and after 12 months (T12) of aging in artificial saliva	**Zipbond Universal** (SDI, Bayswater Vic, Australia)
**Elkaffas, A.A. et al. (2025)** [5]	Sound human molars	80 (10)	dentin pretreatment using 0.015 mol/L copper sulfate (CuSO4) and 0.01 mol/L dipotassium hydrogen phosphate (K2HPO4)	No dentin pretreatment	Immediate testing storage in distilled water at 37 °C for 24 h	**Scotchbond Universal Plus** (3 M Oral Care, Seefeld, Germany)
**Wendlinger, M. et al. (2025)** [3]	Sound human molars	60 (10)	Dentin pretreatment using 50% (v/v) Dimethyl sulfoxide (DMSO) aqueous solution	Untreated dentin surfaces	Specimens were tested at 24 h or after storage in an aqueous solution containing 0.5 chloramine (replaced biweekly) at 37 °C for 4 years	The study used 2 adhesive systems: **1-iBond Universal (IBU)** (Kulzer GmbH) **2- Scotchbond Universal (SBU)** (3 M ESPE, St Paul, MN, USA)
**Ferreira, S. et al. (2025)** [26]	Sound human molars	60 (10)	Selective Dentin Etching (SDE): The dentin surface was pre-treated with 37% phosphoric acid for only three seconds	The study used a Self-Etch (SE) strategy as the control group	Immediate testing storage in distilled water at 37 °C for 24 h	The study used 2 adhesive systems: **1-Optibond Universal (OPU)** (Kerr Corporation, Orange, CA, USA) **2- Futurabond M** + **(FTU)** (VOCOGmbH, Cuxhaven, Germany)
**Öztürk, E. et al. (2025)** [27]	Sound human molars	48 (4)	Selective Dentin Etching (SDE) with 37% phosphoric acid for only three seconds	Compared to the self-Etch (SE) mode and conventional 15-second etching	Immediate testing storage in distilled water at 37 °C for 24 h	The study used 2 adhesive systems: **1- Singlebond Universal adhesive** (3 M ESPE, St. Paul, MN, USA) **2- Adhese Universal (AU)** (Ivoclar Vivadent)
**Liberatori, G.F. et al. (2025)** [10]	Sound human molars	80 (20)	Application of a limonene-containing remineralizing solution as a dentin pretreatment	The study used two control groups: 1- Positive Control (SD): Sound dentin samples that received no pretreatment 2- Negative Control (CAD): Artificially created caries-affected dentin that received no additional treatment	Specimens were tested at 24 h and 6 months of water storage	**Singlebond Universal adhesive** (3 M ESPE, St. Paul, MN, USA)

### Risk of bias assessment of included studies

The JBI & CRIS (Checklist for reporting in vitro studies) guidelines were used to assess the quality of the included studies, following the 11 criteria developed in a previous systematic review of in vitro studies [23]. A detailed description of the evaluated domains is provided in Table 2.

**Table 2:** Risk of bias assessment.

	Use of control group	Standardization of the teeth	Method of sample size calculation	Estimated meaningful difference	Random distribution of samples	Detailed explanation about sample preparation	Samples prepared by single operator	Observer/ outcome assessor blinding	Using characterization approach	Appropriate reporting of statistical analysis	In vitro study stimulating in vivo situations	Risk of Bias
**Stape, T.H.S. et al**. [11]	Yes	Yes	Unclear	Unclear	Yes	Yes	Unclear	Unclear	Yes	Yes	No	Moderate
**Cecchin, D. et al**. [17]	Yes	Yes	Unclear	Unclear	Yes	Yes	Unclear	Unclear	Yes	Yes	No	Moderate
**Zhang, Z. et al**. [14]	Yes	Yes	Unclear	Unclear	Yes	Yes	Unclear	Unclear	Yes	Yes	Yes	Moderate
**Peng, W. et al**. [4]	Yes	Yes	Unclear	Unclear	Yes	Yes	Unclear	Unclear	Yes	Yes	Yes	Moderate
**Fernandes, G.L. et al**. [15]	Yes	Yes	Unclear	Unclear	Yes	Yes	Unclear	Unclear	No	Yes	No	High
**Ahmed, M. et al**. [18]**)**	Yes	Yes	Unclear	Unclear	Yes	Yes	Unclear	Unclear	Yes	Yes	Yes	Moderate
**Mancuso, E. et al**. [13]	Yes	Yes	Unclear	Unclear	Yes	Yes	Unclear	Unclear	Yes	Yes	Yes	Moderate
**Pan, Y. et al**. [16]	Yes	Yes	Unclear	Unclear	Yes	Yes	Unclear	Unclear	Yes	Yes	No	Moderate
**Zhao, S. et al**. [2]	Yes	Yes	Unclear	Unclear	Yes	Yes	Unclear	Unclear	Yes	Yes	Yes	Moderate
**Manihani, A.K. et al**. [6]	Yes	Yes	Unclear	Unclear	Yes	Yes	Unclear	Unclear	Yes	Yes	No	Moderate
**Karadas, M. et al**. [12]	Yes	Yes	Unclear	Unclear	Yes	Yes	Unclear	Unclear	Yes	Yes	Yes	Moderate
**D’ Alessandro, C. et al**. [28]	Yes	Yes	Unclear	Unclear	Yes	Yes	Unclear	Unclear	Yes	Yes	Yes	Moderate
**Elkaffas, A.A. et al**. [5]	Yes	Yes	Yes	Yes	Yes	Yes	Unclear	Yes	No	Yes	No	Low
**Wendlinger, M. et al**. [3]	Yes	Yes	Yes	Yes	Yes	Yes	Unclear	Unclear	Yes	Yes	Yes	Low
**Ferreira, S. et al**. [26]	Yes	Yes	Yes	Yes	Yes	Yes	Unclear	Unclear	Yes	Yes	No	Low
**Öztürk, E. et al**. [27]	Yes	Yes	Unclear	Unclear	Yes	Yes	Unclear	Unclear	Yes	Yes	No	Moderate
**Liberatori, G.F. et al**. [10]	Yes	Yes	Unclear	Unclear	Unclear	Yes	Unclear	Unclear	Yes	Yes	Yes	Moderate

Each study was evaluated by two independent reviewers using these 11 parameters, assigning each item a score of “Yes,” “No,” or “Unclear.” The percentage of “Yes” scores was used to calculate each study’s overall risk of bias: studies with ≤49% were classified as high risk, 50–69% as moderate risk, and ≥70% as low risk. In case of confusion or conflict in the evaluation, a third reviewer was invited to make a judgment [23,24]. The Review Manager (RevMan Version 10.1.1, The Cochrane Collaboration, London, UK) was utilized to produce a summary and graphical illustration of the risk of bias for the selected studies.

### Outcome

The measured outcome was the bond strength (in MPa) of the resin composite to pretreated sound dentin, assessed using the microtensile test.

### Data synthesis

A thematic narrative synthesis was conducted, classifying studies by pretreatment type to gather evidence regarding the influence of chemical surface pretreatments on bond strength to sound dentin. The synthesis was conducted in accordance with the ENTREQ guidelines (Enhancing transparency in reporting the synthesis of qualitative research) [25]. A meta-analysis was proposed but considered impractical due to significant variability in study designs, pretreatment protocols, and testing methodologies, as outlined by PRISMA guidelines [22].

## Results

### Study selection

**Fig. 1:**
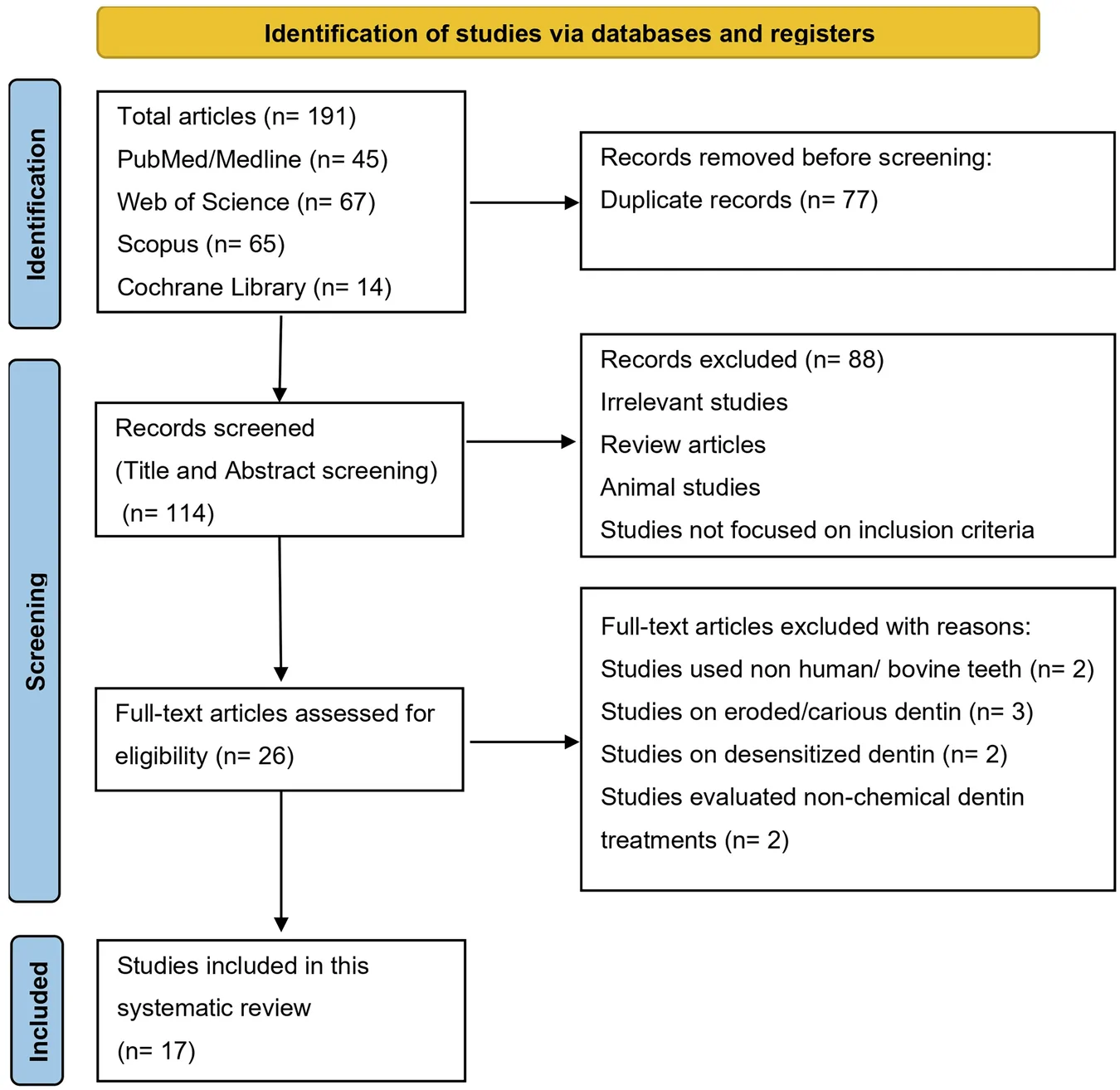
PRISMA flowchart for study selection.

A total of 191 articles were identified through a comprehensive online search of the already mentioned databases. Following the elimination of duplicates, 114 articles proceeded to title and abstract screening. According to the established eligibility criteria, 26 articles were chosen for full-text review. Of these, 17 satisfied the inclusion criteria and were incorporated into this systematic review. The search and selection process is described in the PRISMA flowchart (Fig. 1). Key characteristics of the included studies are summarized in Table 1.

### Description of included studies

All included studies assessed the impact of chemical surface treatments on the dentin of human molars regarding microtensile bond strength. Despite methodological differences across studies, dentin bond strength served as the primary outcome measure in all included studies. The chemical treatments were categorized by their type, and each category’s findings are described individually below.

### Alternative etching strategies

#### Selective dentin etching

Two studies [26,27] of the 17 studies evaluated the effect of selective dentin etching with 37% phosphoric acid for only three seconds, compared with the self-etch mode and traditional 15-second etching. The reduced etching time significantly increased immediate micro-tensile bond strength (µTBS) (*p* < 0.05).

#### Using alternative etchant

Two studies [17,18] of the 17 studies examined the effects of two novel dentin conditioners, glycolic acid (GA) and a metal salt-based conditioner (ZON), as alternatives to traditional phosphoric acid (PA) etching.

Glycolic acid (GA) was tested against phosphoric acid (PA) across three adhesive systems: Adper Single Bond (SB), One Step Plus (OSP), and Scotchbond Universal (SBU). The bond strength of SBU was not significantly affected by the etchant (*p* > 0.05) [17].

An experimental metal salt-based conditioner (ZON) was tested against a traditional 37% phosphoric acid etch-and-rinse (E&R) method and a self-etch (SE) mode. ZON yielded significantly higher immediate µTBS than the conventional etch-and-rinse mode (*p* < 0.05). There was no significant difference between the ZON-pretreated group and the self-etch mode (*p* > 0.05). After aging, no significant differences were observed among the three groups (*p* > 0.05). Additionally, there was no significant reduction in μTBS for ZON and E&R after aging, whereas SE μTBS decreased significantly upon aging [18].

### Solvent-based strategies

#### Resveratrol/ethanol pretreatment

One study of the 17 [4] examined the effects of using four concentrations of a resveratrol/ethanol solution (0, 1, 10, and 20 mg/mL) as a versatile dentin primer to pretreat the dentin surface for 60 s. At 24 hours, all groups showed similar bond strengths (*P* > 0.05). However, following thermocycling and collagenase aging, the groups with 10 and 20 mg/mL resveratrol/ethanol exhibited significantly higher bond strength than the control group (*P* < 0.05).

#### Acetone pretreatment

One study of the 17 [2] assessed the impact of using acetone to pretreat the dentin surface for 60 s. There was no significant difference in immediate µTBS between the ethanol wet-bonding (EWB), acetone wet-bonding (AWB), and water wet-bonding (WWB) groups (*p* > 0.05). However, the 50%AWB group achieved the highest immediate bond strength (*p* < 0.05). Following thermocycling or collagenase aging, the AWB and 50% AWB groups preserved µTBS and showed significantly higher values than the WWB and EWB groups (*p* < 0.05).

#### Dimethyl sulfoxide (DMSO) pretreatment

Four out of 17 studies [3,11,12,14] evaluated the effect of applying a 50% DMSO solution for 60 s before adhesive application. Overall, the results suggested that DMSO may enhance resin–dentin bonding, although this effect varied depending on the adhesive chemistry and experimental conditions. In one study, DMSO/H₂O and DMSO/ethanol pretreatments did not significantly influence the immediate µTBS of the Scotchbond Universal (SU) adhesive (*p* > 0.05) [11].

Another study reported that the combined use of DMSO wet-bonding and epigallocatechin-3-gallate (EGCG) did not significantly affect immediate µTBS compared with the control groups (*p* > 0.05) when Single Bond Universal adhesive was used. However, higher concentrations of EGCG maintained bond strength after aging (*p* > 0.05), producing significantly higher µTBS than those of the other groups (*p* < 0.05) [14].

Additionally, DMSO application significantly increased the µTBS of various universal adhesives, including Clearfil Universal Bond (CUB), Scotchbond Universal (SBU), and Ambar Universal Bond (AUB) (*p* < 0.05), and these positive effects were maintained after aging. However, the bond strength of G-Premio Bond (GPB) was not statistically affected by DMSO application (*p* > 0.05) [12]. In a 4-year study, DMSO-pretreated samples showed no significant reductions in bond strengths after 4 years of water storage (*p* > 0.05). In contrast, groups without DMSO pretreatment exhibited significant reductions in bond strengths (*p* < 0.05) [3].

### Collagen stabilization strategies

#### Chlorhexidine pretreatment

One study assessed the effect of dentin pretreatment with 2% chlorhexidine gluconate (CHX) for 60 seconds. There was no statistically significant difference in immediate µTBS among the groups analyzed (*p* > 0.05). However, the group etched with phosphoric acid without CHX showed the highest numerical bond strength compared with the CHX group tested in self-etch mode [15].

#### Glutaraldehyde pretreatment

One study [13] assessed the effect of pretreating dentin surfaces with two glutaraldehyde-based desensitizers: the liquid GLUMA Desensitizer (L) and the gel GLUMA Desensitizer Powergel (G) for 30 seconds before applying adhesive. Overall, desensitizer application either improved or maintained bond strength, although results varied by formulation and adhesive. Adhese Universal (AU) showed significantly higher bond strength values than iBond Universal (IBU) (*p* < 0.05). Additionally, the liquid version (L) of GLUMA produced significantly higher immediate µTBS than both the gel version (G) and the control groups (*p* < 0.05). After one year of storage in artificial saliva, bond strengths decreased across all groups, but the benefit of pretreatment became even more apparent. Pre-treating dentin with either desensitizer (L or G) resulted in significantly higher bond strength after one year compared to the untreated control groups (*p* < 0.05).

#### Copper-based (Cu-P) pretreatment

Two studies [5,16] of 17 evaluated a novel dentin bonding pretreatment involving a sequential application of 20 µL of copper sulfate (CuSO_4_) and dipotassium hydrogen phosphate (K_2_HPO_4_) solutions for 20 s. In one study, the pretreatment with 0.015 mol/L CuSO_4_ and 0.01 mol/L K_2_HPO_4_ produced highly significant increases in immediate µTBS across all tested groups compared to untreated controls (*p* < 0.05) [5]. In another study [16], the pretreatment was concentration-dependent. The groups with moderate concentrations (H-Cu: 0.15 mol/L CuSO_4_ + 0.1 mol/L K_2_HPO_4_ and L-Cu: 0.015 mol/L CuSO_4_ + 0.01 mol/L K_2_HPO_4_) produced significantly higher immediate µTBS than the untreated control groups (*p* < 0.05). However, the use of a very high concentration (1.5 mol/L CuSO_4_ + 1.0 mol/L K_2_HPO_4_) resulted in significantly lower µTBS than the control (*p* < 0.05). Finally, the lowest concentration tested (0.0015 mol/L CuSO_4_) was found to be ineffective, producing µTBS similar to the control group (*p* > 0.05).

#### Collagen cross-linkers (CCLs) pretreatment

One study [6] investigated the effect of applying two plant-derived CCLs: 6.5% Grape Seed Extract (GSE) and 10% Hesperidin for 5 minutes as a pretreatment to restore bond integrity after contamination. Microtensile results showed that although contamination significantly degrades the bond, both natural cross-linkers successfully restore it to levels near those of the uncontaminated control group. Pretreatment with both CCLs resulted in significantly higher immediate µTBS than in the untreated contaminated group (*p* < 0.05). Moreover, there was no significant difference between the uncontaminated control group and the group treated with Hesperidin (*p* > 0.05).

#### Silver-based pretreatment

One study [28] evaluated the effects of an ammonia-based (Riva Star) and a water-based (Riva Star Aqua) silver-containing solutions as a therapeutic pretreatment for dentin. The protocol involved applying the silver solution for 10 seconds, followed by Potassium Iodide (KI), before applying the universal adhesive. Pretreatment with both solutions significantly reduced immediate µTBS compared with untreated controls (*p* < 0.05). This negative effect persisted after 12 months of aging in artificial saliva (*p* < 0.05).

#### Limonene pretreatment

One study [10] assessed the impact of limonene (LIM) on the bond strength of a universal adhesive system. The pretreatment involved immersing sound dentin samples in a remineralizing solution containing 21 mg/mL of limonene for 2 hours per day over 8 days. Limonene had no significant effect on bond strength, yielding µTBS results comparable to those of the untreated sound dentin controls after 24 h and 6 months of aging.

#### Universal adhesives used in the included studies

A total of 11 universal adhesive systems were evaluated across the included studies. Scotchbond Universal (3 M ESPE, St. Paul, MN, USA) was the most examined, being evaluated in 12 studies. Among the functional monomers, 10-MDP was the most prevalent in these adhesives. Other functional monomers include PENTA (in Prime Bond Universal), GPDM (in Optibond Universal), and MCAP (in Adhese Universal). The detailed compositions of these universal adhesives are summarized in Table 3.

**Table 3:** Composition of universal adhesive systems used in the included studies.

Universal adhesive	Composition*	Included studies
**Scotchbond / Single Bond Universal (SBU)** (3 M ESPE, St Paul, MN, USA)	10-MDP, Bis-GMA, dimethacrylate resins, vitrebond copolymer, HEMA, filler, ethanol, water, initiators, silane.	**Stape, T.H.S. et al**. [11] **Cecchin, D. et al**. [17] **Zhang, Z. et al**. [14] **Peng, W. et al**. [4] **Fernandes, G.L. et al**. [15] **Pan, Y. et al**. [16] **Zhao, S. et al**. [2] **Manihani, A.K. et al**. [6] **Karadas, M. et al**. [12] **Wendlinger, M. et al**. [3] **Öztürk, E. et al**. [27] **Liberatori, G.F. et al**. [10]
**Adhese Universal (AU)** (Ivoclar Vivadent, Schaan, Liechtenstein)	10-MDP, MCAP, HEMA, Bis- GMA, D3MA, Water, Ethanol, highly dispersed silica, Initiators and Stabilizers.	**Ahmed, M. et al**. [18]**)** **Mancuso, E. et al**. [13] **Öztürk, E. et al**. [27]
**iBond Universal (IBU)** (Kulzer GmgH, Hanau, Germany)	10-MDP, UDMA, TEGDMA, 4-methacryloxyethyltrimelliticanhydride, photoinitiators, catalysts, stabilizers, and acetone.	**Mancuso, E. et al**. [13] **Wendlinger, M. et al**. [3]
**Prime Bond Universal (PBU)** (Dentsply Sirona, Konstanz, Germany)	PENTA, Multifunctional acrylate, Water, Isopropanol, Acidic acrylate, Bifunctional acrylate, Camphorquinone, Initiator, Stabilizer.	**Pan, Y. et al**. [16]
**Clearfil Universal Bond (CUB)** (Kuraray Noritake, Okayama, Japan)	10-MDP, Bis-GMA, HEMA, hydrophilic aliphatic dimethacrylate, colloidal silica, silane coupling agent, accelerators, camphorquinone, initiators, ethanol, water.	**Karadas, M. et al**. [12]
**Ambar Universal Bond (AUB)** (FGM, Brazil)	10-MDP, methacrylate monomers, photoinitiators, co-initiators, stabilizers, silica particles, and ethanol.	**Karadas, M. et al**. [12]
**G-Premio Bond (GPB)** (GC Corp., Tokyo, Japan)	10-MDP, 4-MET, MEPS, methacrylate monomers, acetone, water, initiators, silica.	**Karadas, M. et al**. [12]
**Zipbond Universal** (SDI, Bayswater Vic, Australia)	MDP, adhesive monomers, ethanol, water, fluoride, and initiators.	**D’ Alessandro, C. et al**. [28]
**Scotchbond Universal Plus (**3M Oral Care, Seefeld, Germany)	10-MDP, HEMA, 2-propenoic acid, silane, ethanol, water, camphorquinone, a copolymer of acrylic and itaconic acid, and copper (II) acetate monohydrate.	**Elkaffas, A.A. et al**. [5]
**Optibond Universal (OPU)** (Kerr Corporation, Orange, CA, USA)	GPDM, GDMA, HEMA, Acetone, Ethanol.	**Ferreira, S. et al**. [26]
**Futurabond M** + **(FTU)** (VOCOGmbH, Cuxhaven, Germany)	HEMA, Bis-GMA, UDMA, Ethanol, Acidic adhesive monomer, Pyrogenic silica acids.	**Ferreira, S. et al**. [26]

*10-MDP 10-methacryloyloxydecyl dihydrogen phosphate, *Bis-GMA* Bisphenol A diglycidylmethacrylate, *HEMA* 2-Hydroxyethyl methacrylate, *MCAP* Methacrylated carboxylic acid polymer, *D3MA* Decandiol dimethacrylate, *UDMA* Urethane Dimethacrylate, *TEGDMA* Triethylene Glycol Dimethacrylate, *PENTA* dipentaerythritol penta acrylate monophosphate, *4-MET* 4-methacryloxyethyl trimellitic acid, *MEPS* methacryloyloxyalkyl thiophosphate methylmethacrylate, *GPDM* Glycerol phosphate dimethacrylate, *GDMA* Glycerol dimethacrylate.

#### Risk of bias assessment of included studies

**Fig. 2:**
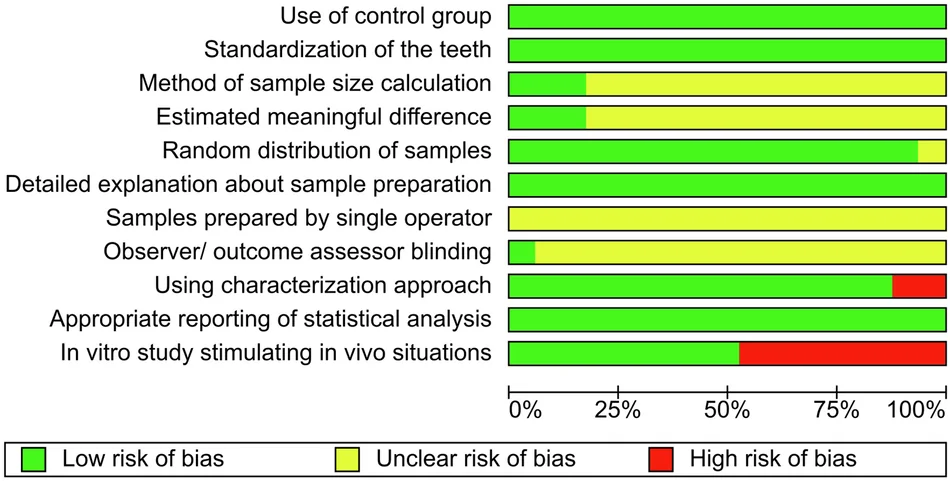
Risk of bias graph.

**Fig. 3:**
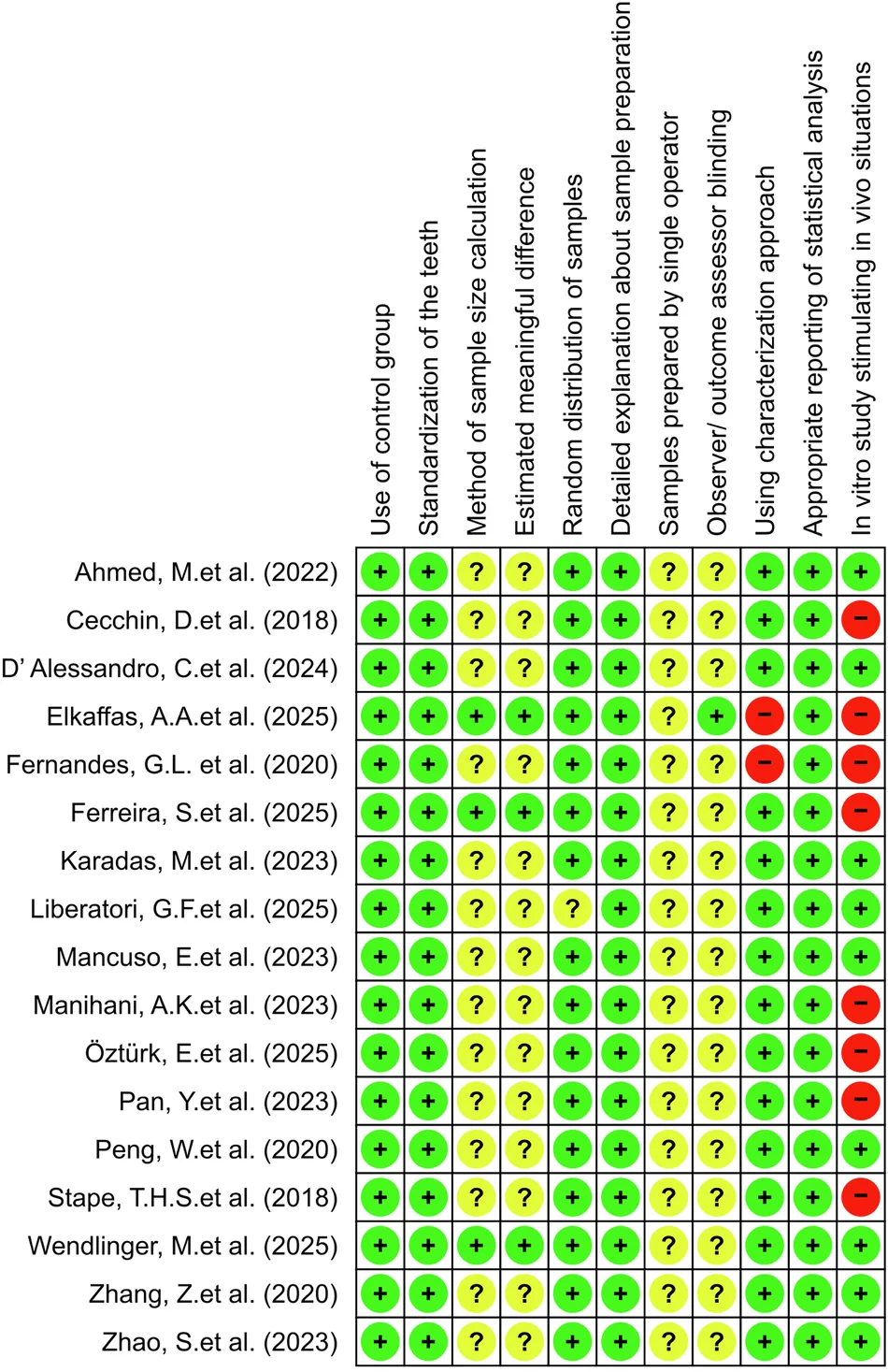
Risk of bias summary.

Based on the parameters specified in the analysis, one study was classified as having a high risk of bias. Three studies were categorized as having a low risk of bias, while the remaining 13 studies demonstrated a moderate risk of bias. The primary methodological bias was ascribed to the lack of a sample size calculation, observer blinding, and the failure of several in vitro studies to simulate in vivo situations. The risk of bias assessment results are clearly presented in Table 2. The corresponding risk of bias graph and summary are illustrated in Figs. 2 and 3, respectively.

## Discussion

Laboratory studies are important for addressing clinical issues, enabling systematic assessment of various treatments and translating positive outcomes into clinical practice. Restorative dentistry now favors conservative, less invasive methods that depend heavily on the efficacy of dental adhesives. Despite advances, bond durability remains limited, and more than 50% of all dental work involves replacing or repairing old composite restorations [2,3]. The main challenge is the gradual biodegradation of the hybrid layer, commonly regarded as the “weak link” of the restorative interface [4]. A complex interplay of mechanical, chemical, and biological factors drives this degradation. Therefore, additional pretreatments have been suggested to improve dentin bonding and extend the lifespan of adhesive restorations. This systematic review was conducted to assess the influence of several chemical dentin pretreatments on the microtensile bond strength of resin composite, based on a comprehensive review of existing in vitro studies.

This systematic review included 17 in vitro studies that collectively evaluated the bond strength of universal adhesives to dentin, depending on the additional chemical pretreatment used: alternative etching strategies, such as selective dentin etching for 3 s and the use of alternative etchants; solvent-based strategies, such as resveratrol/ethanol, acetone, and dimethyl sulfoxide (DMSO) pretreatments; and collagen stabilization strategies, such as chlorhexidine, glutaraldehyde, copper-based (Cu-P), collagen cross-linkers (CCLs), silver-based, and limonene pretreatments. These strategies were systematically assessed to determine their impact on bond strength to resin composite. Many of these techniques enhanced the bond strength of universal adhesives to dentin, while others had no effect and a few impaired it.

Selective dentin etching (SDE) with 37% phosphoric acid for only 3 s significantly improved immediate µTBS compared with self-etch mode and traditional 15 s etching [26,27]. This technique limits the depth of demineralization, enhances surface wettability, and prevents the formation of a zone of denuded, unprotected collagen at the base of the hybrid layer, a primary cause of bond degradation over time. Unlike conventional 15 s etching, which deeply demineralizes dentin (4–5 µm) and removes Hydroxyapatite (HAp) crystals crucial for chemical bonding of functional monomers such as 10-MDP, SDE preserves HAp within the collagen, enabling both micromechanical and chemical bonding [26,27]. These findings align with previous studies [11,29] that also reported improved bond strength with reduced etching time.

Research on new conditioners seeks alternatives to the 37% traditional phosphoric acid etching, which often deeply demineralizes dentin and leaves exposed collagen vulnerable to degradation. Emerging agents such as glycolic acid and Zirconium Oxynitrate (ZON) aim to establish a more stable resin–dentin interface by preserving hydroxyapatite for chemical bonding or providing structural reinforcement through metallic ions. Glycolic acid, an α-hydroxy acid frequently utilized in dermatology, has been investigated as a gentle etchant for dental substrates. Glycolic acid (GA) produced bond strength values similar to those of 35% phosphoric acid (PA) [17]. This finding aligns with previous studies [30–32] that attributed this outcome to variations in the molecular weights of the two acids, which may affect diffusion and substrate demineralization. Glycolic acid has a lower molecular weight (76.05 g/mol) compared to phosphoric acid (97.99 g/mol) [31]. Furthermore, the acid dissociation constant (pKa) that influences the demineralization potential of both solutions is relatively strong; the pKa of PA is 2.16, whereas the pKa of GA is 3.83 [31]. This enables GA to efficiently modify the etching pattern to enhance bonding.

Zirconium Oxynitrate (ZON) is a new metal salt-based conditioner with calcium-chelating properties. One study [18] reported that ZON achieved markedly superior immediate microtensile bond strength compared with phosphoric acid etching and remained stable over 50,000 thermocycles. In contrast to conventional etchants, ZON forms a hybrid layer that integrates micro-mechanical interlocking at the surface with chemical adhesion of functional monomers (such as 10-MDP) to residual hydroxyapatite beneath. Furthermore, ZON leaves dentinal tubules nearly closed with fewer resin tags, which is supposed to minimize postoperative hypersensitivity [18].

Solvent choice is crucial for bonding universal adhesives. Solvents displace water, improve curing, and keep hydrophobic monomers in solution, preventing phase separation. Multiple studies have used ethanol, acetone, and Dimethyl Sulfoxide (DMSO) to optimize the resin–dentin interface by managing moisture, boosting resin infiltration, and enhancing hybrid layer stability. One study [4] demonstrated that higher concentrations of resveratrol/ethanol improved μTBS values after aging, owing to resveratrol’s role as a dentin primer. Resveratrol, a natural polyphenol with three phenolic hydroxyl groups, bio-modifies collagen via hydrogen bonding. Ethanol was used as a solvent because of resveratrol’s limited water solubility. Ethanol can enhance collagen cross-linking by reducing the dielectric constant of the medium [33], implying its potential to augment the modifying action of resveratrol on collagen. This cross-linking fortifies the collagen scaffold against enzymatic degradation and thermal stress. However, future clinical trials are essential to validate the efficacy of resveratrol/ethanol primers.

For the acetone wet-bonding (AWB) technique, one study [2] showed that pretreating dentin with acetone or a 50% aqueous acetone solution enhanced immediate bond strength. This improvement was maintained even after 10,000 thermocycles and 1 month of collagenase aging. This is because acetone expands the collagen matrix, allowing deeper adhesive penetration. Acetone possesses the highest vapor pressure among dental solvents, enabling it to quickly eliminate excess moisture from the demineralized matrix [34]. This inhibits the formation of water-filled voids that undermine bond strength. Furthermore, acetone has a lower viscosity than ethanol, facilitating superior penetration of demineralized dentin [35]. Additionally, the amphiphilic nature of acetone enables rapid displacement and evaporation of residual water, delivering both hydrophobic (MDP) and hydrophilic (HEMA) monomers into the spaces between the expanded collagen fibrils [35]. This enhanced monomer infiltration and water substitution reduce exposed collagen and enzymatic breakdown, improving bond durability.

The impact of Dimethyl Sulfoxide (DMSO) pretreatment was assessed in four studies [3,11,12,14]. Overall, the results showed that DMSO enhanced bond strength to air-dried dentin and maintained this benefit after aging, although the impact varied with adhesive chemistry and experimental conditions [12]. Many studies highlight DMSO’s favorable effects on resin-dentin bonding, explained by its capacity to re-expand dried collagen, enabling better adhesive penetration [36–39]. It has a higher hydrogen bonding force (δh) than air-dried collagen, potentially breaking interpeptide hydrogen bonds that cause shrinkage and restoring diffusion pathways for resin infiltration [11]. Furthermore, DMSO efficiently displaces remaining water from the demineralized matrix, exposing more binding sites for chemical interaction, especially for 10-MDP [3,14]. However, DMSO did not improve the bond strength of G-Premio Bond (GPB) [12]. Researchers attributed this to its high solvent concentration and severe acidity, which may compromise the mechanical properties of the adhesive layer. In a long-term study, DMSO maintained bond strength over 4 years of water storage [3]. Additionally, incorporating natural biomodifiers such as 0.1% or 1 wt% epigallocatechin-3-gallate (EGCG) further enhanced stability by preventing µTBS reduction after collagenase aging [14]. This stability is attributed to DMSO-induced water displacement, along with EGCG’s activity as a powerful inhibitor of matrix metalloproteinases (MMPs) and its antibacterial impact against S. mutans [14].

For chlorhexidine pretreatment, one study [15] found that preapplication of 2% chlorhexidine gluconate (CHX) did not significantly affect the immediate bond strength of the universal adhesive system. The researchers explained that chlorhexidine possesses a strong cationic charge that binds to anionic phosphate in hydroxyapatite, possibly forming precipitates that act as a physical barrier, reducing contact between the adhesive and the tooth. It may also physically block dentinal tubules with residual debris, hindering resin infiltration, as the CHX was not rinsed off. These results only cover immediate bond strength; CHX’s main benefit is suppressing matrix metalloproteinases (MMPs) for long-term bond stability, which was not tested here [15].

The effect of pretreating dentin surfaces with a glutaraldehyde-based desensitizer was assessed in one study [13]. Overall, the results showed that its use either improved or maintained bond strength. Contributing factors included the desensitizer’s components: 2-hydroxyethyl methacrylate (HEMA), which lowers water surface tension and aids deeper resin monomer penetration into the tubules [40,41]. The desensitizer also has a pH of about 3 and acts as a mild self-etching agent that partially dissolves the smear layer without the hazards associated with phosphoric acid etching. This exposes tubule orifices for improved micromechanical interlocking, while hydroxyapatite residues remain available for chemical bonding [42]. Glutaraldehyde and HEMA can form a layer with collagen, which chemically interacts with the adhesive resin, resulting in a copolymerization effect that reinforces the bond [43,44].

Additionally, the liquid desensitizer produced significantly higher immediate µTBS than both the gel formulation and the untreated control groups. The researchers suggested that the gel’s higher viscosity may hinder its diffusion into the dentin collagen fibrils and smear layer, in contrast to the more flowable liquid [13]. Previous studies have noted potential cytotoxicity of glutaraldehyde with HEMA, especially when applied directly to demineralized dentin. Using it on mineralized, less permeable dentin is safer, but case-specific evaluations are needed [45,46].

Copper-based pretreatment (Cu-P) using copper sulfate (CuSO4) and dipotassium hydrogen phosphate (K_2_HPO_4_) was introduced in two studies [5,16]. The results showed that this pretreatment improved the bond strength of universal adhesives, especially at 0.015-0.15 mol/L CuSO4, by producing copper phosphate nanoparticles that support collagen, enhance wettability, and facilitate better resin infiltration [5,16]. Furthermore, copper is essential for the activity of the collagen cross-linking enzyme lysyl oxidase, thereby strengthening collagen, acting as a cross-linker, and inhibiting Matrix Metalloproteinase-2 (MMP-2) [5,16,47,48]. However, the effectiveness of Cu-P pretreatment was concentration-dependent. At 1.5 mol/L CuSO_4_, bond strength decreased significantly. This is because excessive precipitates form rapidly, blocking dentin tubules and hindering the infiltration of adhesive monomers. When the concentration was too low at 0.0015 mol/L, the solution was too dilute to modify the dentin surface or improve bond strength [16].

For collagen cross-linkers (CCLs), one study [6] evaluated the effectiveness of 6.5% proanthocyanidin (grape seed extract (GSE)) and 10% hesperidin in restoring bond strength after blood and hemostatic agent contamination. The results revealed that GSE improved bond strength more than contaminated samples but less than controls, likely because proanthocyanidin forms hydrogen bonds with proteins, inhibits collagenase, and converts soluble collagen into an insoluble form [6]. A previous systematic review identified GSE as the most effective natural cross-linker for maintaining bond strength post-aging [49]. Additionally, hesperidin yielded results comparable to controls, likely due to its lower molecular weight and stronger collagen binding, thereby inhibiting collagen degradation [50–52].

Using antimicrobial materials like silver diamine fluoride (SDF) offers a drill-free approach to arrest dental caries, with SDF providing protection against MMPs and cysteine cathepsins (CCs) [53–55]. However, it causes unesthetic dark staining due to residual silver ions reacting with organic material [56,57]. To reduce staining, applying potassium iodide (KI) over ammonia-based SDF (Riva Star) has been proposed. One study [28] found that silver-containing solutions like Riva Star decreased the bond strength of universal adhesives after one year. This outcome was attributed to silver salts forming precipitates that block resin infiltration and reduce ion availability crucial for chemical bonding [58,59]. The high alkalinity of silver solutions can also impair the etching process, and water-based Riva Star Aqua may leave residual water, affecting monomer polymerization and leading to hydrolytic degradation [58,60]. While some studies have supported the use of SDF + KI before bonding procedures [61], others have not [59,62], likely due to varied methodologies, application protocols, and product differences [63].

Natural substances are being studied to improve the stability of dental adhesive interfaces and extend the longevity of restorations. Their benefits and minimal side effects compared with synthetic agents drive this interest [64,65]. One study [10] evaluated the effect of limonene (LIM) pretreatment on the bond strength of a universal adhesive system. Results showed that LIM-treated sound dentin had bond strength similar to untreated dentin at both aging times, consistent with the findings of Garcia Pinto et al. [66]. Limonene likely removes the smear layer, exposing dentinal tubules and aiding resin infiltration for stable adhesion [66]. It also helps arrest early caries progression by redepositing Ca and P ions, inhibiting collagen degradation [67], and preventing bacterial growth, adhesion, and acid production [68]. Additionally, limonene may act as a protease inhibitor, neutralizing oxidants and reducing bacterial acid production [67]. In contrast to previous pretreatments applied for seconds to minutes, this protocol required a daily immersion of 2 hours over 8 days, indicating considerable heterogeneity among the included treatments.

The evaluated universal adhesives contain various functional monomers that influence bonding performance. 10-MDP is the most common and more durable than other monomers due to its superior chemical bonding and stability [3,11]. In addition to 10-MDP, other functional monomers include GPDM, PENTA, and 4-MET. Furthermore, Polyalkenoic Acid Copolymers (PAC), or the Vitrebond copolymer, form ionic bonds with calcium, aiding adhesion, but may compete with 10-MDP for calcium-binding sites and weaken bonds [5,27]. Therefore, variations in the functional monomers should be considered when interpreting the effects of the investigated chemical pretreatments, as the adhesive formulation itself may contribute to the variability observed among the included studies.

The risk of bias affected the robustness of the evidence. In terms of quality evaluation of the relevant studies, the majority (13 studies) demonstrated a moderate risk of bias, rendering them vulnerable to certain biases; however, this is likely inadequate to invalidate the results. The moderate risk of bias was primarily due to the absence of a sample size calculation and the lack of blinding. A restricted sample size generally diminishes study power, complicates the estimation of significant differences, and increases the likelihood of type II errors, or false negatives [23]. The absence of blinding may result in an overestimation of treatment effects [69]. Also, the lack of thermocycling in certain studies limited the applicability of the findings. As a result, while several chemical pretreatments demonstrated promising benefits for dentin bond strength, these findings should be regarded with caution until they are verified by well-designed research employing more precise methodology.

Finally, this systematic review has several limitations that should be considered in future studies. First, it included only in vitro studies, due to the limited number of relevant clinical studies addressing the review question. As a result, the findings may not be directly applicable to clinical practice because laboratory research cannot fully replicate the oral environment. Another limitation is the exclusion of non-English studies because of access issues and a lack of translated sources, which narrowed the scope of the review. Furthermore, the review focused only on studies conducted on sound human dentin, excluding enamel and carious or sclerotic dentin. Additionally, a meta-analysis was not possible because of substantial variation in pretreatment techniques, limiting the ability to quantitatively synthesize results. Therefore, to validate the results of the current review, more research, including randomized clinical trials, is needed.

## Conclusion

Within the limitations of this systematic review, the in vitro evidence suggests that universal adhesives, when paired with specific chemical pretreatments, may improve bonding to sound dentin. Selective Dentin Etching and milder etchants, such as Glycolic Acid and Zirconium-based conditioner (ZON), can match or outperform phosphoric acid while inducing less extensive dentin demineralization. Acetone wet bonding, DMSO pretreatment, glutaraldehyde desensitizers, and copper-based (Cu-P) pretreatment showed promising results in improving bond strength. Collagen cross-linkers, such as hesperidin and GSE, help restore bond strength after contamination with blood or hemostatic agents. Silver-based solutions should be avoided, as they can impair bond strength. However, further studies are needed to confirm these findings.

## Supplementary information

Search strategy in different databases

PRISMA 2020 Checklist

## Data Availability

The data supporting the findings of this study are available from the corresponding author upon reasonable request.
