# Supplementary material for: Effect of chemical surface pretreatments on microtensile bond strength of resin composite to dentin when using universal adhesives: a systematic review of laboratory studies
Source: BDJ Open. 2026 Oct 20;12:101. doi: 10.1038/s41405-026-00484-3 (PMC13683406; doi:10.1038/s41405-026-00484-3)
Supplement: Supplementary file 1 [file 41405_2026_484_MOESM1_ESM.docx]

**Supplementary file.1:** Search strategy in different databases

| **Database** | **Search keywords** |
| --- | --- |
| **PubMed** | (("Dentin" OR "dentin") AND ("Surface Conditioning" OR "surface treatment" OR "pretreatment" OR "etching" OR "acid treatment") AND ("tensile bond strength" OR "microtensile bond strength") AND ("Universal Adhesives" OR "universal adhesive" OR "multi-mode adhesive")) AND ("in vitro" OR "laboratory") NOT (review) |
| **Web of Science** | ((((AB=(Dentin)) AND AB=(pretreatment)) AND AB= (microtensile bond strength)) AND AB= (Universal Adhesives)) NOT TS=(review) |
| **Scopus** | TITLE-ABS (Dentin ) AND TITLE-ABS (pretreatment) AND TITLE-ABS (microtensile bond strength) AND TITLE-ABS (Universal Adhesives) AND PUBYEAR > 2012 AND PUBYEAR < 2027 |
| **Cochrane library** | (Dentin pretreatment) AND (microtensile bond strength) AND (Universal Adhesives) |
